# Dynamic liver dysfunction predicts poor survival in patients with EGFR-mutant non-small cell lung cancer and liver metastases treated with EGFR tyrosine kinase inhibitors

**DOI:** 10.1186/s12885-026-15616-z

**Published:** 2026-01-23

**Authors:** Wen Zhang, Xuemei Wu, Xiaorong Sun, Jian Wen, Xiaoli He, Mingzhou Zhang, Guansong Wang, Zhi Xu

**Affiliations:** Institute of Respiratory Diseases, Department of Pulmonary and Critical Care Medicine, Xinqiao Hospital, Army Medical University, 183 Xinqiao Street, 400037 Chongqing, China

**Keywords:** EGFR mutation, Non-small cell lung cancer, Liver metastasis, Tyrosine kinase inhibitor, Liver function, Prognostic biomarker, Dynamic monitoring

## Abstract

**Background:**

Liver metastasis is an adverse prognostic factor in patients with epidermal growth factor receptor (EGFR)-mutant non-small cell lung cancer (NSCLC). While baseline liver burden is a known risk factor, the prognostic significance of dynamic liver function changes during targeted therapy remains understudied. This study aimed to evaluate the prognostic value of Consecutive Liver Function Abnormalities (CLFA) in this population.

**Methods:**

We retrospectively analyzed 82 patients with EGFR-mutant NSCLC and liver metastases receiving first-line EGFR-TKIs. CLFA was defined as the presence of any abnormal liver function parameter (ALT, AST, ALP, GGT, TBIL, or ALB) at three consecutive time points (baseline, 6 weeks, and 12 weeks). Metabolic confounders and hepatic tumor burden were evaluated. To address immortal time bias and distinguish dynamic deterioration from baseline impairment, time-dependent Cox regression, landmark analysis (12-week), and sensitivity analysis (excluding patients with baseline abnormalities) were performed.

**Results:**

CLFA was identified in 31 patients (37.8%). Baseline metabolic characteristics were balanced between groups. Hepatotoxicity was predominantly mild (CTCAE Grade 1–2), with no dose reductions mandated by liver injury. Patients with CLFA had significantly shorter overall survival (OS) (median 20.4 vs. 34.3 months, Log-rank *P* < 0.001) and liver-specific progression-free survival (Log-rank *P* = 0.038). In multivariate analysis adjusting for potential confounders, including liver metastatic burden, CLFA remained an independent prognostic marker for poor OS (HR 2.56, 95% CI 1.25–5.24, *P* = 0.010). This association was robustly confirmed by time-dependent Cox regression (HR 2.61, *P* = 0.010) and landmark analysis (*P* = 0.0017). Notably, in a sensitivity analysis restricted to patients with normal baseline liver function, the subsequent development of CLFA was still associated with a two-fold increase in mortality risk (HR 2.03), indicating a deleterious effect of acquired liver injury independent of baseline status.

**Conclusions:**

CLFA serves as a robust, dynamic prognostic marker independent of baseline tumor burden or initial liver function. The acquisition of persistent low-grade liver dysfunction signifies an aggressive clinical trajectory associated with significantly inferior survival, distinct from acute hepatotoxicity. These findings underscore the value of CLFA for risk stratification beyond standard safety monitoring.

**Supplementary Information:**

The online version contains supplementary material available at 10.1186/s12885-026-15616-z.

## Introduction

Liver metastasis occurs in approximately 20–30% of patients with advanced non–small cell lung cancer (NSCLC) and is associated with a distinctively poor prognosis [1–3]. While epidermal growth factor receptor (EGFR) tyrosine kinase inhibitors (TKIs) have significantly improved survival, their therapeutic efficacy is often attenuated in patients with hepatic involvement [4, 5]. This reduced efficacy likely results from the complex interplay between hepatic tumor burden and compromised liver function reserve.

Liver function abnormalities are frequently encountered during EGFR-TKI treatment. These may arise from drug-induced hepatotoxicity, tumor infiltration, or metabolic stress [6–9]. While transient enzyme elevations are often clinically insignificant, persistent abnormalities may signal ongoing hepatic injury or reduced metabolic capacity. Previous studies have primarily focused on baseline liver dysfunction or the mere presence of metastases [4, 10, 11]. However, the prognostic value of dynamic liver function changes during targeted therapy remains poorly defined.

We propose that Consecutive Liver Function Abnormality (CLFA)—representing sustained biochemical disturbance rather than isolated elevations—offers superior prognostic insight. Persistent hepatic dysfunction may alter drug metabolism and reflect underlying hepatic vulnerability in patients with liver metastases. Despite the known adverse outcomes associated with liver metastases [12, 13], few studies have specifically evaluated CLFA and its relationship with different generations of EGFR-TKIs.

Therefore, this study evaluated the prognostic significance of CLFA in patients with EGFR-mutant NSCLC and liver metastases receiving first-line EGFR-TKIs. By integrating longitudinal biochemical data with survival outcomes, we sought to determine whether sustained liver function abnormalities serve as a dynamic biomarker for risk stratification in this high-risk population.

## Methods

### Study design and patients

This retrospective cohort study included patients with histologically confirmed stage IV EGFR-mutant non-small cell lung cancer (NSCLC) and liver metastases who received first-line EGFR-TKI monotherapy at Xinqiao Hospital, Army Medical University, between January 2020 and March 2025. Patient eligibility was defined by the completion of liver function tests at three specific time points (baseline, 6 weeks, and 12 weeks during TKI treatment) and the availability of imaging studies with measurable disease assessable for treatment response. Patients were excluded if they had: (1) chronic viral hepatitis or cirrhosis; (2) concomitant use of non–cancer-related hepatotoxic agents; or (3) incomplete clinical or laboratory follow-up. The study was conducted in accordance with the Declaration of Helsinki and was approved by the Institutional Review Board of Xinqiao Hospital, Army Medical University (approval number: 2025-199-01), with the requirement for informed consent waived due to its retrospective nature.

### Data collection and definitions

Clinical data, including age, sex, smoking history, ECOG performance status (PS), EGFR mutation subtype, histology, TKI generation, and baseline organ involvement, were extracted from electronic medical records. To account for potential hepatic confounders, we collected detailed data on Body Mass Index (BMI), alcohol consumption history, and metabolic comorbidities. Metabolic syndrome was defined according to the NCEP ATP III criteria [14], requiring the presence of at least three of the following five risk factors: abdominal obesity, elevated triglycerides, reduced HDL cholesterol, elevated blood pressure, and elevated fasting glucose.

Patients received EGFR-TKIs (Gefitinib 250 mg qd, Erlotinib 150 mg qd, Icotinib 125 mg tid, Afatinib 40 mg qd, Osimertinib 80 mg qd, Almonertinib 110 mg qd, or Furmonertinib 80 mg qd) orally. The initial dose was prescribed according to standard manufacturer guidelines. Reduced starting doses were not routinely mandated for patients with mild-to-moderate baseline liver dysfunction unless accompanied by other contraindications (e.g., poor performance status, advanced age, or low body weight).

The usage of hepatoprotective drugs was documented throughout the treatment period. In this study, hepatoprotective agents primarily included glutathione, polyene phosphatidylcholine, magnesium isoglycyrrhizinate, and bicyclol, which were administered either prophylactically or therapeutically at the discretion of the treating physician. Dose modifications (reductions or interruptions) were recorded to assess tolerability.

### Hepatic tumor burden and liver function assessment

Baseline hepatic tumor burden was quantified according to RECIST version 1.1 [15]. We recorded the number of liver metastases (categorized as < 3 vs. ≥3), the sum of the longest diameters (SLD) of measurable hepatic target lesions, the maximum diameter of the largest hepatic lesion, and the distribution of liver metastases (unilobar vs. bilobar).

Liver function indices, including alanine aminotransferase (ALT), aspartate aminotransferase (AST), alkaline phosphatase (ALP), γ-glutamyl transferase (GGT), total bilirubin (TBIL), and albumin (ALB), were recorded at baseline and at each treatment cycle. The severity of liver function abnormalities was graded according to the Common Terminology Criteria for Adverse Events (CTCAE) version 5.0 [16].

### Definition of consecutive liver function abnormality (CLFA)

Consecutive Liver Function Abnormality (CLFA) was defined as the occurrence of at least one abnormal liver function test parameter at each of three consecutive assessment time points: baseline, 6 weeks, and 12 weeks after the initiation of TKI therapy. An abnormal result was defined as a value exceeding the institutional upper limit of normal (ULN) for ALT, AST, ALP, GGT, and TBIL, or a value below the lower limit of normal (< 35 g/L) for ALB. Notably, abnormalities were not required to involve the same specific biochemical parameter across consecutive assessments; the persistent presence of any abnormal liver function result at all three time points was sufficient to define the CLFA phenotype.

### Outcome definitions

Progression-free survival (PFS) was defined as the time from treatment initiation to disease progression at any site or death from any cause. Overall survival (OS) was defined as the time from treatment initiation to death from any cause. Liver-specific PFS (Liver-PFS) was defined as the time from the initiation of EGFR-TKI therapy to the date of first documented disease progression within the liver or death. Liver progression was assessed according to RECIST version 1.1 [15], defined as: (1) ≥ 20% increase in the sum of diameters of hepatic target lesions; (2) unequivocal progression of hepatic non-target lesions; or (3) appearance of new hepatic metastatic lesions. Radiologic review was performed by investigators and confirmed by radiologic reports.

### Statistical analysis

Continuous variables were compared using the Student’s t-test or Mann–Whitney U test, and categorical variables using the χ² or Fisher’s exact test. Longitudinal changes in liver function parameters were assessed via the Friedman test. Due to strict inclusion criteria, there were no missing data for the primary definition of CLFA. Minimal missing baseline data (smoking status, *n* = 3) were handled using mode imputation.

Survival outcomes were estimated using the Kaplan–Meier method and compared via the log-rank test. Multivariable Cox proportional hazards models included variables with *P* < 0.10 in univariate analysis or strong clinical relevance. To avoid multicollinearity among hepatic tumor burden metrics, “Number of Liver Metastases” was selected as the representative covariate for the final model. Potential metabolic confounders (BMI, alcohol, diabetes, etc.) were rigorously evaluated but excluded from the final model due to lack of significance.

Model robustness was verified through several approaches: (1) Collinearity was ruled out using the Variance Inflation Factor (all VIF < 2.0); (2) The Proportional Hazards (PH) assumption was confirmed via Schoenfeld residuals; (3) Immortal time bias was addressed using a landmark analysis at 12 weeks; and (4) The dynamic nature of liver function was modeled using time-dependent Cox regression. Subgroup analyses employed Likelihood Ratio Tests for interactions, with P-values adjusted for multiple comparisons using the Benjamini-Hochberg False Discovery Rate (FDR) method. All analyses were performed using R software (version 4.5.1).

## Results

### Baseline characteristics of the study population

**Fig. 1:**
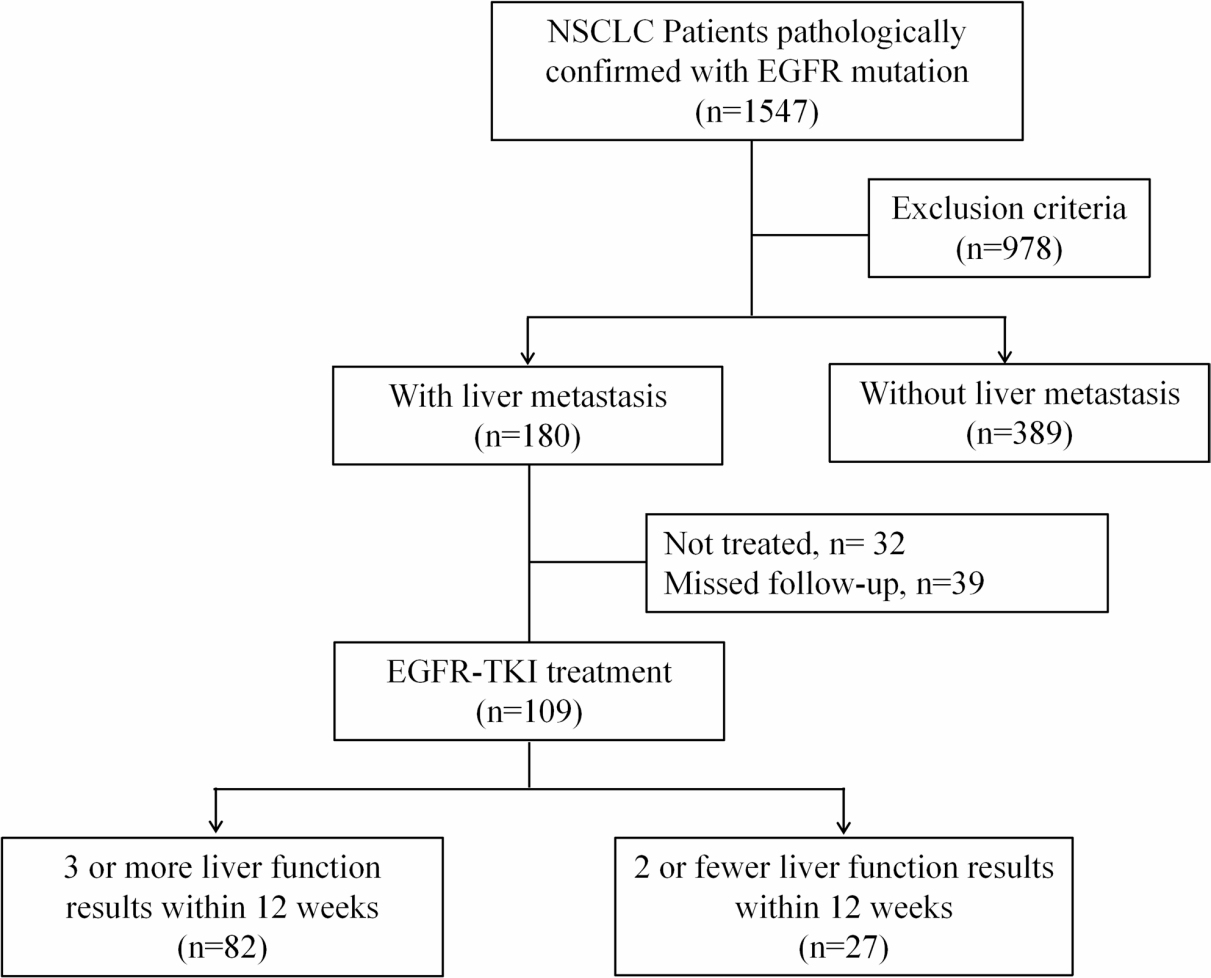
Flowchart of patient selection. A total of 1,547 patients with pathologically confirmed EGFR-mutant NSCLC were initially screened. After applying exclusion criteria, 180 patients were identified with liver metastases. Patients were further excluded if they did not receive treatment (*n* = 32) or were lost to follow-up (*n* = 39). Among the 109 patients treated with EGFR-TKIs, those with insufficient liver function monitoring (defined as ≤ 2 assessments within the first 12 weeks) were excluded. The final study cohort consisted of 82 patients with complete longitudinal data

A total of 82 patients with EGFR-mutant NSCLC and liver metastases were included (Fig. 1). Based on longitudinal liver function monitoring, 31 patients (37.8%) were classified into the CLFA group, while 51 patients (62.2%) were in the Non-CLFA group. Baseline characteristics are summarized in Table 1. The two groups were well-balanced regarding age, sex, smoking history, ECOG performance status, histology, EGFR mutation subtype, and the presence of brain or bone metastases (all *P* > 0.05). Notably, to address potential metabolic confounders, we evaluated BMI, alcohol consumption, diabetes, NAFLD, and metabolic syndrome, finding no statistically significant differences between the two groups (all *P* > 0.05). However, patients in the CLFA group exhibited a trend toward a higher burden of liver metastases (≥ 3 lesions: 74.2% vs. 47.1%; *P* = 0.051) and presented with significantly higher baseline liver enzyme levels and lower albumin compared to the Non-CLFA group (*P* < 0.05). Consequently, the usage of hepatoprotective drugs was significantly higher in the CLFA group (22.6% vs. 2.0%; *P* = 0.007).

**Table 1:** Baseline characteristics of patients with EGFR-mutant NSCLC and liver metastases

Variable	All patients (*n* = 82)	CLFA group (*n* = 31)	Non-CLFA group (*n* = 51)	*p*-value
Demographic data				
Age, years	57.4 ± 10.1	55.7 ± 9.1	58.5 ± 10.6	0.209
Male, n (%)	32 (39.0%)	16 (51.6%)	16 (31.4%)	0.112
Anthropometrics & Comorbidities				
BMI	22.7 ± 3.3	22.9 ± 3.8	22.6 ± 3.3	0.703
Alcohol consumption, n (%)	7 (6.4%)	4 (12.9%)	3 (5.9%)	0.487
Diabetes, n (%)	8 (7.3%)	2 (6.5%)	6 (11.8%)	0.687
NAFLD, n (%)	9 (8.2%)	1 (3.2%)	8 (15.7%)	0.166
Metabolic syndrome, n (%)	4 (3.6%)	1 (3.2%)	3 (5.9%)	0.990
Hepatic Tumor Burden				
SLD, cm (Median, IQR)	3.0 (1.8–5.3)	3.5 (2.0-6.3)	2.9 (1.8–4.4)	0.184
Largest hepatic lesion diameter, cm (Median, IQR)	1.8 (1.3–2.5)	1.8 (1.4–2.6)	1.8 (1.2–2.3)	0.233
Bilobar liver involvement, n (%)	48 (43.6%)	20 (64.5%)	28 (54.9%)	0.531
Number of liver metastases, n (%)				0.051
<3	35 (42.7%)	8 (25.8%)	27 (52.9%)	
≥3	47 (57.3%)	23 (74.2%)	24 (47.1%)	
Clinical Characteristics				
Smoking history, n (%)	19 (23.2%)	9 (29.0%)	10 (19.6%)	0.508
ECOG performance status, n (%)				0.999
0–1	62 (75.6%)	24 (77.4%)	38 (74.5%)	
≥2	18 (22.0%)	7 (22.6%)	11 (21.6%)	
Histology (adenocarcinoma vs. others)	79/3	30/1	49/2	0.999
The usage of hepatoprotective drugs, n (%)	8 (9.6%)	7 (22.6%)	1 (2.0%)	0.007
Tumor characteristics				
EGFR mutation subtype, n (%)				0.663
Exon 19 deletion	37 (45.1%)	15 (48.4%)	22 (43.1%)	
Exon 21 L858R	34 (41.5%)	11 (35.5%)	23 (45.1%)	
Others	11 (13.4%)	5 (16.1%)	6 (11.8%)	
Brain metastases, n (%)	49 (59.8%)	22 (71.0%)	27 (52.9%)	0.167
Bone metastases, n (%)	60 (73.2%)	24 (77.4%)	36 (70.6%)	0.674
Treatment characteristics				
EGFR-TKI generation, n (%)				0.798
1st/2nd generation	29 (35.4%)	12 (38.7%)	17 (33.3%)	
3rd generation	53 (64.6%)	19 (61.3%)	34 (66.7%)	
Baseline liver function, (median [IQR])				
ALT (U/L)	18.1 (13.1–25.0)	23.6 (14.2–37.5)	15.9 (12.9–22.7)	0.006
AST (U/L)	20.6 (16.2–26.0)	25.0 (17.8–34.0)	19.8 (15.8–23.4)	0.005
Total bilirubin (µmol/L)	11.0 (9.3–13.0)	10.9 (8.9–12.9)	11.5 (9.4–12.9)	0.860
Albumin (g/L)	41.5 (39.0-44.8)	39.1 (37.3–41.8)	42.8 (41.2–45.1)	0.001
ALP (U/L)	96.4 (76.0-123.4)	152.0 (104.3-274.5)	86.1 (65.5-100.5)	< 0.001
GGT (U/L)	31.9 (22.3–61.3)	66.7 (34.6-114.8)	26.4 (19.0-34.3)	< 0.001
Outcome follow-up				
Follow-up time, months (Median, IQR)	20.0 (11.2–30.1)	15.9 (8.0-21.3)	23.0 (12.5–35.2)	0.009

Data Presentation: Data are presented as mean ± standard deviation (SD) for normally distributed continuous variables, median (interquartile range [IQR]) for non-normally distributed variables, and number (percentage) for categorical variables

Statistical Analysis: P-values were calculated using the Student’s t-test, Mann-Whitney U test, Chi-square test, or Fisher’s exact test, as appropriate

*Abbreviations* *SLD* the sum of the longest diameters, *ALT* alanine aminotransferase, *AST* aspartate aminotransferase, *ALP* alkaline phosphatase, *BMI* Body Mass Index, *CLFA* Consecutive Liver Function Abnormalities, *ECOG* Eastern Cooperative Oncology Group, *EGFR* epidermal growth factor receptor, *GGT* gamma-glutamyl transferase, *IQR* interquartile range, *NAFLD* Non-alcoholic Fatty Liver Disease, *NSCLC* non-small cell lung cancer, *TKI* tyrosine kinase inhibitor

Regarding treatment administration, the vast majority of patients (80/82, 97.6%) initiated EGFR-TKI therapy at the standard dose. Only two patients (one in each group) received a reduced starting dose of afatinib (30 mg), both due to advanced age and low body weight to mitigate potential mucocutaneous or gastrointestinal toxicity, rather than baseline hepatic impairment.

### Dynamic changes in liver function and safety profile

The longitudinal dynamics of liver function parameters during the first 12 weeks of EGFR-TKI therapy are detailed in Table 2. Statistically significant dynamic elevations were observed for ALT, AST, and GGT from baseline to Cycle 2 (Friedman test, *P* < 0.05), reflecting treatment-related hepatic stress. GGT and ALP were the most frequent drivers of the CLFA phenotype, with continuous abnormalities observed in 30.5% and 21.0% of patients, respectively.

**Table 2:** Dynamic changes of liver function parameters during EGFR-TKI treatment

Parameters		Baseline (T0)	Cycle 1 (T1)	Cycle 2 (T2)	*p*-value	Max CTCAE Grade Grade 1–2	Max CTCAE Grade Grade ≥ 3	Continuous abnormality, *n* (%)
ALT (U/L)		18.1 (13.1–25.0)	24.5 (15.9–37.5)	24.8 (18.1–44.1)	< 0.001	27 (32.5%)	0 (0%)	11 (13.4%)
AST (U/L)		20.6 (16.2–26.0)	24.0 (19.1–39.0)	26.2 (21.1–34.8)	< 0.001	26 (31.3%)	1 (1.2%)	9 (11.0%)
TBIL (µmol/L)	11.0 (9.3–13.0)		10.0 (7.7–12.4)	10.6 (8.5–13.4)	0.401	9 (11.0%)	0 (0%)	1 (1.2%)
GGT (U/L)		31.9 (22.3–61.3)	33.4 (22.1–66.8)	36.8 (24.9–68.1)	0.008	33 (39.8%)	3 (3.6%)	25 (30.5%)
ALP (U/L)		96.4 (76.0-123.4)	96.1 (70.5-124.4)	87.2 (65.0-140.3)	< 0.001	30 (36.1%)	4 (4.8%)	17 (21.0%)
ALB (g/L)		41.5 (39.0-44.8)	38.8 (35.0-42.6)	41.5 (36.8–44.5)	< 0.001	21 (25.3%)	0 (0%)	11 (17.7%)
Patients with CLFA, n (%)		—	—	—		—	—	31 (37.8%)

Data Presentation: Continuous variables are presented as median (interquartile range [IQR]), and categorical variables are presented as number (percentage)

Statistical Analysis: P-values were calculated using the Friedman test to assess the statistical significance of dynamic changes across the three time points (Baseline, Cycle 1, and Cycle 2)

Toxicity Grading: Toxicity grades were defined according to the Common Terminology Criteria for Adverse Events (CTCAE) version 5.0. “Max CTCAE Grade” represents the highest grade observed for each patient across the monitored time points

Definitions: CLFA (Consecutive Liver Function Abnormalities) was defined as the presence of abnormal values for any specific parameter at all three time points (T0, T1, and T2)

*Abbreviations* *ALT* alanine aminotransferase, *AST* aspartate aminotransferase, *TBIL* total bilirubin, *GGT* gamma-glutamyl transferase, *ALP* alkaline phosphatase, *ALB* albumin, *ULN* upper limit of normal, *LLN* lower limit of normall, *CLFA* consecutive liver function abnormality

Despite these biochemical fluctuations, the overall severity of hepatotoxicity was mild. The vast majority of liver function abnormalities were CTCAE Grade 1–2. The incidence of severe hepatotoxicity (Grade ≥ 3) was remarkably low (1.2% for AST, 3.6% for GGT, 4.8% for ALP). Dose reductions were documented in only 5 patients, all attributable to non-hepatic toxicities (diarrhea, *n* = 3; rash, *n* = 2). Importantly, no dose reductions or treatment interruptions were mandated specifically by hepatotoxicity, confirming the generally good hepatic tolerability of the regimen.

### Survival outcomes according to CLFA status

**Fig. 2:**
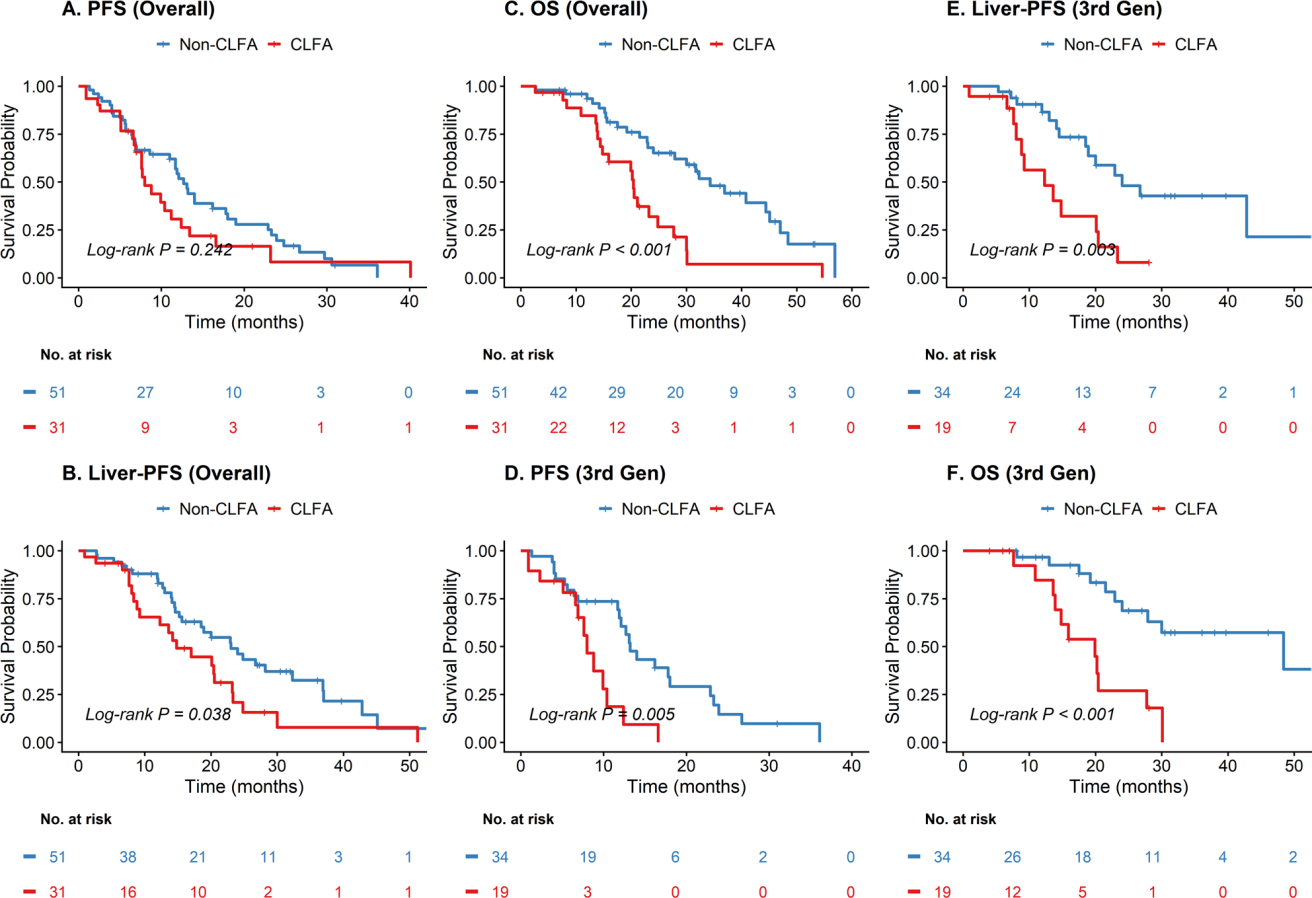
Kaplan-Meier survival estimates stratifying patients by Consecutive Liver Function Abnormalities (CLFA) status. **A–C** Analysis in the overall population: **A** Progression-Free Survival (PFS), **B** Liver-specific Progression-Free Survival (Liver-PFS), and (**C**) Overall Survival (OS). **D–F** Subgroup analysis in patients treated with third-generation EGFR-TKIs: **D** PFS, **E** Liver-PFS, and **F** OS. Patients with CLFA (red line) demonstrated consistently inferior survival outcomes compared to the Non-CLFA group (blue line) across all endpoints. P-values were calculated using the two-sided log-rank test. Abbreviations: CLFA, Consecutive Liver Function Abnormalities; EGFR-TKI, Epidermal Growth Factor Receptor Tyrosine Kinase Inhibitor; OS, Overall Survival; PFS, Progression-Free Survival

At a median follow-up of 20.0 months, 47 deaths were recorded. Patients in the Non-CLFA group demonstrated significantly superior survival outcomes compared to the CLFA group (Fig. 2). The median Overall Survival (OS) was 34.3 months in the Non-CLFA group versus 20.4 months in the CLFA group (Log-rank *P* < 0.001). Similarly, Liver-specific PFS was significantly prolonged in the Non-CLFA group (median 23.0 vs. 14.8 months; Log-rank *P* = 0.038). Although the difference in median PFS did not reach statistical significance in the overall cohort (12.7 vs. 8.0 months; Log-rank *P* = 0.242), stratifying by TKI generation revealed that non-CLFA patients treated with third-generation EGFR-TKIs achieved significantly better PFS (13.2 vs. 8.0 months, Log-rank *P* = 0.005) and OS (48.4 vs. 19.9 months, Log-rank *P* < 0.001) compared to their CLFA counterparts (Fig. 2D–F).

To rigorously exclude the potential confounding effect of immortal time bias (as the definition of CLFA requires survival up to 12 weeks), a landmark analysis was performed by setting a landmark time at 12 weeks. Patients who died or experienced disease progression within the first 12 weeks were excluded. As shown in Supplementary Fig. 1, the negative prognostic trend of CLFA remained consistent. Patients in the CLFA group demonstrated significantly inferior OS compared with the Non-CLFA group (Log-rank *P* = 0.0017). Although the difference in PFS did not reach statistical significance in this reduced landmark cohort (Log-rank *P* = 0.34), the separation of the survival curves supports the robustness of CLFA as a long-term prognostic marker independent of early attrition bias.

### Multivariate and time-dependent cox regression

In multivariate Cox regression analysis adjusting for age, ECOG PS, EGFR mutation subtype, TKI generation, and number of liver metastases, CLFA remained an independent predictor of poor OS (Adjusted HR 2.56, 95% CI 1.25–5.24, *P* = 0.010) (Table 3). Assessment of the proportional hazards assumption using Schoenfeld residuals confirmed that CLFA satisfied the assumption in both PFS (*P* = 0.126) and OS (*P* = 0.938) models (Supplementary Fig. 2).

**Table 3:** Univariate and multivariate Cox regression analyses for progression-free survival (PFS) and overall survival (OS)

Variables	PFS – Univariate analysis		PFS – Multivariate analysis		OS – Univariate analysis		OS – Multivariate analysis	
	HR (95% CI)	p-value	HR (95% CI)	*p*-value	HR (95% CI)	*p*-value	HR (95% CI)	*p*-value
Continuous liver function abnormality (CLFA vs. non-CLFA)	1.37 (0.81–2.33)	0.244	1.50 (0.81–2.78)	0.195	2.72 (1.48-5.00)	0.001	2.56 (1.25–5.24)	0.010
Age (< 65 vs. ≥ 65 years)	0.61 (0.33–1.11)	0.104	0.41 (0.21–0.78)	0.006	0.93 (0.46–1.88)	0.832	0.92 (0.45–1.90)	0.829
Sex (male vs. female)	0.86 (0.51–1.46)	0.585	0.67 (0.38–1.18)	0.166	1.19 (0.66–2.16)	0.561	0.89 (0.47–1.66)	0.705
ECOG PS (≥ 2 vs. 0–1 )	5.14 (2.68–9.87)	< 0.001	9.18 (4.30-19.59)	< 0.001	2.11 (1.09–4.10)	0.027	2.89 (1.38–6.04)	0.005
EGFR mutation type (21 L858R vs. 19 Del)	1.82 (1.08–3.05)	0.024	1.72 (0.98–3.03)	0.059	1.71 (0.95–3.08)	0.076	1.37 (0.74–2.53)	0.319
Type of EGFR-TKI (3rd vs. 1st/2nd generation)	1.01 (0.61–1.69)	0.959	0.67 (0.38–1.19)	0.175	0.70 (0.39–1.27)	0.239	0.51 (0.26–0.98)	0.043
Number of liver metastases (≥ 3 vs. < 3)	1.40 (0.84–2.34)	0.202	1.54 (0.89–2.66)	0.126	2.12 (1.13–3.97)	0.019	2.54 (1.29-5.00)	0.007
NAFLD (yes vs. no)	0.71 (0.33–1.52)	0.382	-	-	0.69 (0.29–1.65)	0.407	-	-
BMI	1.03 (0.96–1.11)	0.357	-	-	1.02 (0.94–1.11)	0.565	-	-
History of alcohol consumption (yes vs. no)	0.75 (0.27–2.10)	0.589	-	-	1.33 (0.47–3.74)	0.588	-	-
Diabetes (yes vs. no)	1.05 (0.47–2.32)	0.905	-	-	1.26 (0.56–2.84)	0.574	-	-
Metabolic syndrome (yes vs. no)	0.63 (0.22–1.81)	0.392	-	-	0.61 (0.19–1.98)	0.410	-	-
Presence of brain metastasis (yes vs. no)	1.26 (0.75–2.12)	0.384	-	-	1.78 (0.97–3.28)	0.063	-	-
Presence of bone metastasis (yes vs. no)	1.38 (0.78–2.44)	0.263	-	-	1.41 (0.74–2.68)	0.292	-	-
Baseline AST (> ULN vs. ≤ ULN)	1.67 (0.71–3.91)	0.241	-	-	1.78 (0.62–5.10)	0.282	-	-
Baseline ALT (> ULN vs. ≤ ULN)	2.00 (0.85–4.73)	0.115	-	-	2.03 (0.79–5.22)	0.140	-	-
Baseline albumin (< 35 vs. ≥ 35 g/L)	1.79 (0.55–5.81)	0.329	-	-	3.22 (0.97–10.65)	0.056	-	-
Baseline bilirubin (> ULN vs. ≤ ULN)	0.79 (0.25–2.54)	0.693	-	-	1.13 (0.35–3.68)	0.834	-	-
Liver metastases pattern (Multiple vs. Solitary)	1.24 (0.71–2.15)	0.452	-	-	1.84 (0.93–3.64)	0.080	-	-
SLD (per cm)	1.17 (1.07–1.28)	< 0.001	-	-	1.13 (1.03–1.24)	0.012	-	-
Largest Hepatic Lesion Diameter (per cm)	1.29 (1.10–1.51)	0.002	-	-	1.38 (1.13–1.69)	0.002	-	-
Bilobar Involvement (Yes vs. No)	1.10 (0.66–1.85)	0.706	-	-	1.27 (0.69–2.31)	0.442	-	-
CLFA (Time-Dependent)*	-	-	1.42 (0.72–2.77)	0.312	-	-	2.61 (1.25–5.44)	0.010

Variables Inclusion: Variables with a P-value < 0.10 in univariate analysis or those with strong clinical relevance (age, sex, ECOG PS, EGFR mutation type, TKI generation, and number of liver metastases) were entered into the multivariate Cox regression model

Tumor Burden: Detailed tumor burden metrics (SLD, largest lesion diameter and liver metastases pattern) were excluded from the multivariate model to avoid multicollinearity with the “Number of liver metastases”

Exclusions: Metabolic factors (BMI, NAFLD, alcohol consumption, diabetes, metabolic syndrome) were evaluated in univariate analysis but were excluded from the final multivariate model due to a lack of statistical significance (*P* > 0.10)

*Abbreviations* *CI* confidence interval, *CLFA* consecutive liver function abnormalities, *ECOG PS* Eastern Cooperative Oncology Group performance status, *EGFR* epidermal growth factor receptor, *HR* hazard ratio, *OS* overall survival, *PFS* progression-free survival, *TKI* tyrosine kinase inhibitor, *ULN* upper limit of normal, *SLD* the sum of the longest diameters, *NAFLD* Non-alcoholic Fatty Liver Disease

*: Time-dependent Cox regression analysis. CLFA was modeled as a time-varying covariate to account for the dynamic nature of liver function and potential immortal time bias. The status was defined as time-dependent, with the exposure window starting at 12 weeks post-treatment initiation. This model was also adjusted for the full set of covariates listed above

To address the dynamic nature of liver function and potential immortal time bias, we further performed a time-dependent Cox regression analysis, treating CLFA as a time-varying covariate. After multivariable adjustment, the development of CLFA remained an independent prognostic factor for both PFS (Time-dependent HR = 1.42, 95% CI 0.72–2.77, *P* = 0.312) and OS (Time-dependent HR = 2.61, 95% CI 1.25–5.44, *P* = 0.010) (Table 3). This confirms that the prognostic value of CLFA for overall survival is driven by dynamic biological changes during treatment rather than solely by baseline characteristics.

### Subgroup analysis and interaction tests

**Fig. 3:**
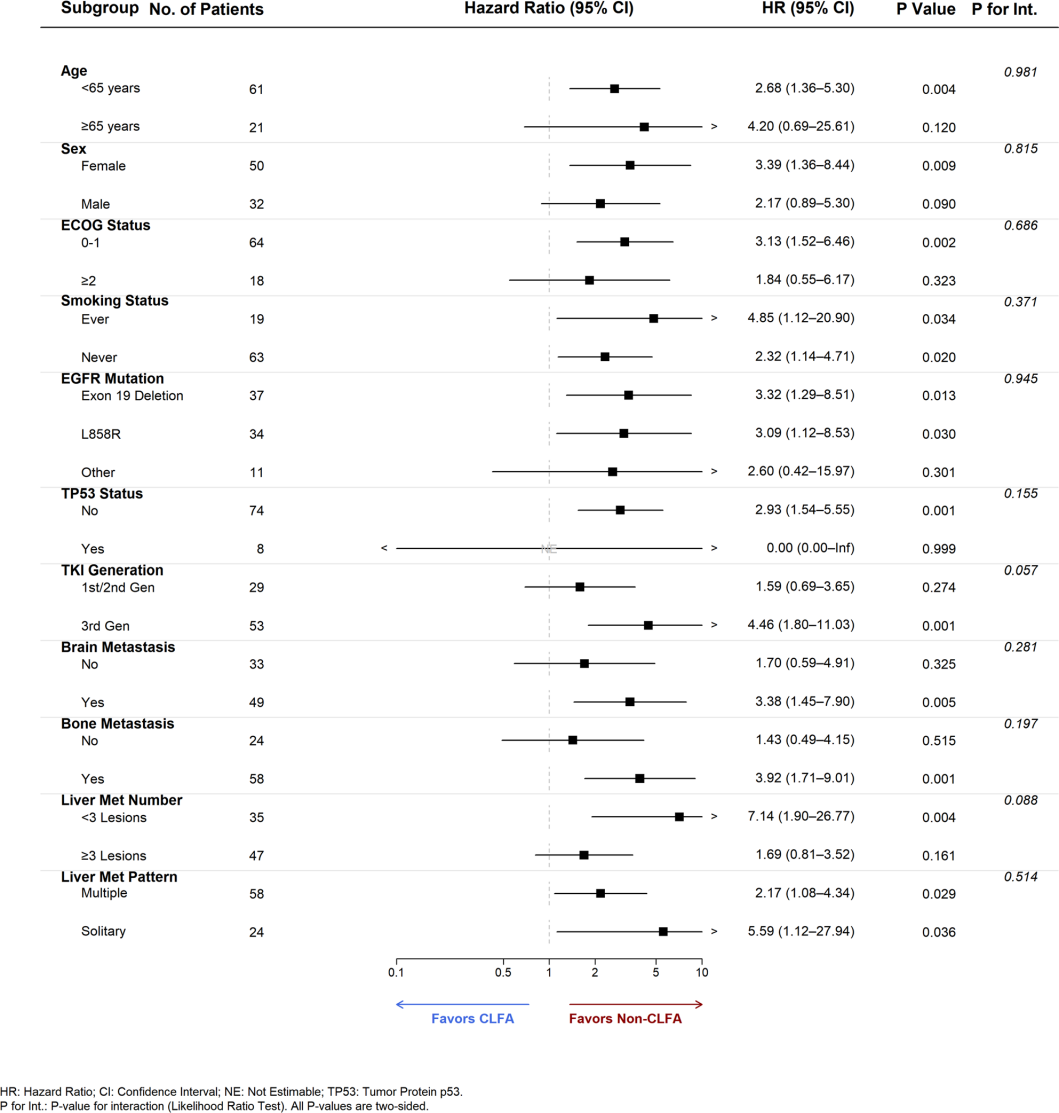
Forest plot of subgroup analysis for Overall Survival (OS) stratified by Consecutive Liver Function Abnormalities (CLFA) status. The forest plot displays the Hazard Ratios (HRs) and 95% Confidence Intervals (CIs) for the association between CLFA and Overall Survival across key clinical subgroups. Estimates were derived from unstratified Cox proportional hazards models within each subgroup. Interpretation: The vertical dashed line at HR = 1.0 represents the line of no effect. An HR > 1.0 (to the right of the dashed line) indicates a higher risk of death for patients with CLFA (favoring the Non-CLFA group), whereas an HR < 1.0 indicates a lower risk (favoring the CLFA group). Statistics: P Value indicates the two-sided significance level for the association within each specific subgroup. P for Int. represents the P-value for interaction between the subgroup variable and CLFA status, calculated using the Likelihood Ratio Test to assess effect heterogeneity. Note: The hazard ratio for the TP53 co-mutation “Yes” subgroup is marked as NE (Not Estimable) due to the limited sample size and lack of events in the reference group, which prevented reliable model convergence

Subgroup analyses were conducted to evaluate the consistency of CLFA’s prognostic value across key clinical strata (Fig. 3; Table 4). The negative impact of CLFA on OS was remarkably consistent across the majority of subgroups. Specifically, CLFA was significantly associated with inferior OS in patients aged < 65 years (HR 2.68, *P* = 0.004), females (HR 3.39, *P* = 0.009), patients with ECOG PS 0–1 (HR 3.13, *P* = 0.002), and those without TP53 co-mutations (HR 2.93, *P* = 0.001). To control for multiple comparisons, we applied the Benjamini-Hochberg False Discovery Rate (FDR) correction. The associations between CLFA and poor OS remained robustly significant or maintained strong trends in these key subgroups after FDR adjustment (Adjusted *P* < 0.05).

**Table 4:** Subgroup analyses of the association between consecutive liver function abnormality (CLFA) and survival outcomes

Subgroup	No.	PFS			OS			*p* -value for Interaction^b^
		Events	HR (95% CI)	*p*-value^a^	Events	HR (95% CI)	*p*-value^a^	(PFS/OS)
Age								0.626/0.981
<65 years	61	49	1.48 (0.82–2.66)	0.195	37	2.68 (1.36–5.30)	0.004*	
≥65 years	21	14	0.95 (0.25–3.62)	0.944	10	4.20 (0.69–25.61)	0.120	
Sex								0.815/0.815
Male	32	25	1.22 (0.54–2.78)	0.630	22	2.17 (0.89–5.30)	0.090	
Female	50	38	1.60 (0.76–3.38)	0.215	25	3.39 (1.36–8.44)	0.009*	
ECOG PS								0.938/0.686
0–1	64	47	1.47 (0.79–2.72)	0.224	33	3.13 (1.52–6.46)	0.002*	
≥2	18	16	1.52 (0.53–4.34)	0.434	14	1.84 (0.55–6.17)	0.323	
Smoking status								0.250/0.371
Never-smoker	63	48	1.23 (0.67–2.28)	0.506	34	2.32 (1.14–4.71)	0.020*	
Ever-smoker	19	15	2.35 (0.66–8.30)	0.186	13	4.85 (1.12–20.90)	0.034	
EGFR mutation type								0.862/0.997
Exon 19 deletion	37	30	1.68 (0.75–3.75)	0.208	23	3.32 (1.29–8.51)	0.013*	
Exon 21 L858R	45	33	1.37 (0.65–2.87)	0.404	24	2.71 (1.15–6.36)	0.022*	
TP53 Status								0.768/0.155
Yes	8	7	1.18 (0.23–5.90)	0.844	4	NE^c^	1.000	
No	74	56	1.36 (0.77–2.40)	0.284	43	2.93 (1.54–5.55)	0.001*	
Type of EGFR-TKI								0.057/0.057
1st/2nd generation	29	26	0.80 (0.35–1.81)	0.590	26	1.59 (0.69–3.65)	0.274	
3rd generation	53	37	2.87 (1.34–6.16)	0.007*	21	4.46 (1.80–11.03)	0.001*	
Number of liver metastases								0.150/0.088
≥3	47	38	0.94 (0.49–1.81)	0.854	31	1.69 (0.81–3.52)	0.161	
<3	35	25	3.19 (1.14–8.95)	0.028*	16	7.14 (1.90–26.77)	0.004*	
Liver metastases pattern								0.582/0.514
Multiple	58	45	1.21 (0.66–2.23)	0.538	35	2.17 (1.08–4.34)	0.029	
Solitary	24	18	2.20 (0.66–7.36)	0.202	12	5.59 (1.12–27.94)	0.036	
Baseline ALT (U/L)								0.911/0.963
<50	75	57	1.22 (0.69–2.18)	0.492	42	2.50 (1.32–4.77)	0.005*	
Baseline AST (U/L)								0.489/0.285
<40	75	57	1.21 (0.68–2.15)	0.524	43	2.49 (1.31–4.72)	0.005*	
Presence of brain metastasis								0.001*/0.281
Yes	49	36	3.68 (1.73–7.85)	< 0.001*	28	3.38 (1.45–7.90)	0.005*	
No	33	27	0.40 (0.14–1.17)	0.095	19	1.70 (0.59–4.91)	0.325	
Presence of bone metastasis								0.293/0.371
Yes	60	45	1.72 (0.91–3.26)	0.094	32	3.51 (1.60–7.71)	0.002*	
No	22	18	0.98 (0.35–2.81)	0.977	15	1.71 (0.56–5.21)	0.342	

*Abbreviations CI* confidence interval, *CLFA* consecutive liver function abnormalities, *HR* hazard ratio, *NE* not estimable, *FDR* false discovery rate, *ALT* alanine aminotransferase, *AST* aspartate aminotransferase, *ECOG PS* Eastern Cooperative Oncology Group performance status

a: P-values calculated using unstratified Cox proportional hazards models comparing CLFA vs. Non-CLFA groups within each subgroup

b: P-values for interaction were calculated using likelihood ratio tests. Nominal (unadjusted) P-values are presented. After applying the Benjamini-Hochberg (FDR) correction, the interaction for brain metastasis in PFS remained statistically significant (Padj < 0.05)

c: NE: Not estimable due to small sample size or lack of events in the subgroup

*: Indicates nominal statistical significance (*P* < 0.05). Notably, after applying the Benjamini-Hochberg (FDR) correction, the strong associations with Overall Survival remained robustly significant (Padj < 0.05), whereas the significance for a PFS subgroup (liver metastasis < 3) was attenuated due to sample size limitations

Regarding PFS, significant interactions were observed. A statistically significant interaction was identified between CLFA and brain metastases (P for interaction = 0.001). The detrimental effect of CLFA on PFS was strongly pronounced in patients with brain metastases (HR 3.68, 95% CI 1.73–7.85, *P* < 0.001), whereas no significant prognostic impact was observed in those without (HR 0.40, *P* = 0.095).

Notably, regarding hepatic tumor burden, CLFA significantly stratified risk in patients with a lower metastatic burden (< 3 liver lesions), showing high Hazard Ratios for both PFS (HR 3.19, *P* = 0.028) and OS (HR 7.14, *P* = 0.004). This suggests that CLFA identifies a high-risk subset even among patients with theoretically limited liver disease.

We also observed notable findings regarding treatment type and tumor burden. In patients treated with third-generation EGFR-TKIs, CLFA was strongly associated with both shorter PFS (HR 2.87, *P* = 0.007) and OS (HR 4.46, *P* = 0.001), with a borderline interaction for PFS (P for interaction = 0.057). Regarding hepatic tumor burden, CLFA significantly stratified risk in patients with a lower metastatic burden (< 3 liver lesions), showing high Hazard Ratios for both PFS (HR 3.19, *P* = 0.028) and OS (HR 7.14, *P* = 0.004). This suggests that CLFA identifies a high-risk subset even among patients with theoretically limited liver disease.

### Sensitivity analysis for dynamic evaluation

To rigorously exclude the confounding effect of baseline impairment, a sensitivity analysis was performed restricted to patients with normal baseline liver function (*n* = 51). In this subgroup, patients who subsequently developed acquired abnormalities showed a clinically substantial trend towards inferior survival compared to those who remained stable. The Hazard Ratio for OS was 2.03 (95% CI: 0.84–4.90, *P* = 0.110), and for PFS was 1.69 (95% CI: 0.86–3.32, *P* = 0.120) (Supplementary Fig. 3). Consistent results were observed in the landmark analysis at 12 weeks (OS HR = 2.05). Although statistical significance was attenuated by the reduced sample size, the magnitude of the hazard ratio (HR > 2.0) provides compelling evidence that dynamic, on-treatment liver injury conveys independent prognostic information beyond baseline characteristics.

## Discussion

The management of EGFR-mutant NSCLC with liver metastases remains a substantial clinical challenge. Despite the transformative efficacy of EGFR-TKIs, hepatic involvement is consistently associated with inferior survival outcomes across treatment settings [13, 17–19]. In this study, we demonstrate that consecutive liver function abnormalities (CLFA), defined as the presence of any abnormal liver biochemical parameter at each of three consecutive assessments within the first 12 weeks of therapy, represent an independent prognostic marker of poor outcome. Patients who developed CLFA experienced significantly shorter OS and liver-PFS. These associations remained robust after adjustment for liver metastatic burden, baseline liver biochemistry, and other clinically relevant confounders.

Unlike prior studies focusing on baseline liver dysfunction or high-grade hepatotoxicity requiring treatment modification [20–23], our findings highlight the prognostic relevance of dynamic, low-grade, but sustained liver dysfunction early during treatment. Mild biochemical abnormalities are common during EGFR-TKI therapy and are often overlooked when they do not mandate dose adjustment [7, 24, 25]. Our results indicate that such abnormalities, when persistent, convey meaningful prognostic information beyond baseline disease characteristics.

Liver metastasis has been consistently identified as a negative prognostic factor in EGFR-mutant NSCLC [1, 2, 4, 13], yet outcomes within this population remain heterogeneous. Our analysis adds granularity by identifying a functional phenotype: patients who maintained stable liver function during early therapy achieved significantly better survival than those who developed CLFA. This finding aligns with prior evidence linking organ function, treatment tolerance, and survival in advanced NSCLC [26–28], and extends these observations to a dynamic, on-treatment context. The relatively low prevalence of smokers in our cohort (23.2%) is consistent with the established epidemiology of EGFR-mutant NSCLC, which is enriched in never-smokers and East Asian populations. Large meta-analyses and real-world cohorts have reported smoking rates of approximately 20–35% among EGFR-mutant patients [29–31], supporting the representativeness of our study population regarding this demographic feature.

A critical question raised by our findings is whether CLFA acts as a direct biological contributor to inferior survival or primarily serves as a surrogate marker for aggressive disease biology and extensive hepatic tumor burden. We acknowledge that patients who developed CLFA presented with a higher baseline liver metastatic burden, consistent with prior studies linking tumor load to liver dysfunction [13, 18, 32]. However, several lines of evidence suggest CLFA captures a distinct clinical trajectory rather than merely reflecting baseline severity. First, CLFA remained independently associated with survival after multivariable adjustment for quantitative tumor burden. Second, to rigorously distinguish the impact of treatment-emergent liver abnormalities from baseline impairment, we conducted a sensitivity analysis excluding patients with baseline abnormalities. Notably, even among patients who started with normal liver function, the subsequent development of CLFA was associated with a clinically substantial two-fold increase in the risk of death (HR 2.03) compared to those who remained stable. Although the statistical significance of this specific subgroup analysis was attenuated by the limited sample size, the magnitude of the hazard ratio provides compelling evidence that dynamic, on-treatment liver injury conveys independent prognostic information beyond baseline characteristics. Finally, to address potential immortal time bias, we validated these findings using landmark analysis and time-dependent Cox regression in the full cohort, both of which confirmed the robustness of the association.

The mechanisms underlying the association between CLFA and poor survival remain speculative. The liver is central to EGFR-TKI metabolism via cytochrome P450 enzymes, particularly CYP3A4, and impaired liver function may influence drug exposure or systemic tolerance even in the absence of overt hepatotoxicity [33–35]. In addition, hepatic metastases are associated with metabolic and inflammatory disturbances that may adversely affect host resilience [36, 37]. This biological rationale may also explain the interpretation of PFS versus OS. While CLFA may not immediately compromise the initial radiological response to TKIs (reflected in PFS), the cumulative hepatic dysfunction limits the host’s physiological reserve to tolerate subsequent lines of therapy. This limitation accelerates systemic deterioration and shortens post-progression survival, thereby driving the pronounced impact on Overall Survival. Accordingly, CLFA should be interpreted as a prognostic marker reflecting the systemic host-tumor interaction, rather than merely a predictor of initial treatment efficacy.

Clinically, liver function tests are inexpensive and routinely available. Early identification of CLFA may serve as a pragmatic warning signal, prompting closer surveillance. Although our study does not support a specific intervention strategy, patients with CLFA may represent an enriched population for future trials exploring intensified or combinatorial strategies, including anti-angiogenic combinations or liver-directed therapies [38, 39].

Several limitations warrant acknowledgment. First, as a retrospective, single-center study, selection bias cannot be fully excluded, although multivariable, landmark, and sensitivity analyses were performed to mitigate this risk. Second, while we adjusted for the number and maximal diameter of liver metastases, volumetric or percentage liver involvement data were unavailable, limiting precise quantification of hepatic tumor burden. Third, pharmacokinetic data were not collected, precluding direct assessment of the relationship between CLFA and EGFR-TKI plasma exposure. Finally, the pragmatic definition of CLFA requires external validation in larger, multi-center cohorts.

In conclusion, CLFA is a simple, dynamic, and clinically accessible prognostic marker in EGFR-mutant NSCLC with liver metastases. Persistent liver function abnormalities early during therapy identify a subgroup with significantly inferior survival, independent of baseline tumor burden. These findings underscore the value of dynamic liver function monitoring beyond safety surveillance and warrant prospective validation.

## Conclusion

Our study establishes CLFA as an independent prognostic marker in EGFR-mutant NSCLC with liver metastases. Crucially, its prognostic value is not merely a surrogate for baseline disease severity; the dynamic acquisition of liver dysfunction, even in patients with normal baseline liver function, elevates mortality risk. Unlike high-grade toxicity that necessitates dose modification, CLFA manifests as a persistent pattern associated with shortened overall survival and an aggressive disease course despite standard therapy. Consequently, routine liver function monitoring provides critical prognostic information beyond safety surveillance, aiding in the early identification of high-risk patients.

## Supplementary Information

Supplementary Material 1.

Supplementary Material 2.

Supplementary Material 3.

## Data Availability

The datasets generated and analyzed during the current study are not publicly available due to patient privacy protection and institutional policy. De-identified data may be made available from the corresponding author upon reasonable request.

## Abbreviations

ALB: Albumin
ALP: Alkaline phosphatase
ALT: Alanine aminotransferase
AST: Aspartate aminotransferase
BMI: Body mass index
CI: Confidence interval
CLFA: Consecutive liver function abnormality
CTCAE: Common terminology criteria for adverse events
ECOG PS: Eastern cooperative oncology group performance status
EGFR: Epidermal growth factor receptor
FDR: False discovery rate
GGT: Glutamyl transferase
HDL: High-density lipoprotein
HR: Hazard ratio
Liver-PFS: Liver-specific progression-free survival
NCEP ATP III: National cholesterol education program adult treatment panel III
NSCLC: Non-small cell lung cancer
OS: Overall survival
PFS: Progression-free survival
PH: Proportional hazards
RECIST: Response evaluation criteria in solid tumors
SLD: Sum of the longest diameters
TBIL: Total bilirubin
TKI: Tyrosine kinase inhibitor
ULN: Upper limit of normal
VIF: Variance inflation factor
