# Supplementary figures and images for: Dynamic liver dysfunction predicts poor survival in patients with EGFR-mutant non-small cell lung cancer and liver metastases treated with EGFR tyrosine kinase inhibitors
Source: BMC Cancer. 2026 Jan 23;26:270. doi: 10.1186/s12885-026-15616-z (PMC12917993; doi:10.1186/s12885-026-15616-z)

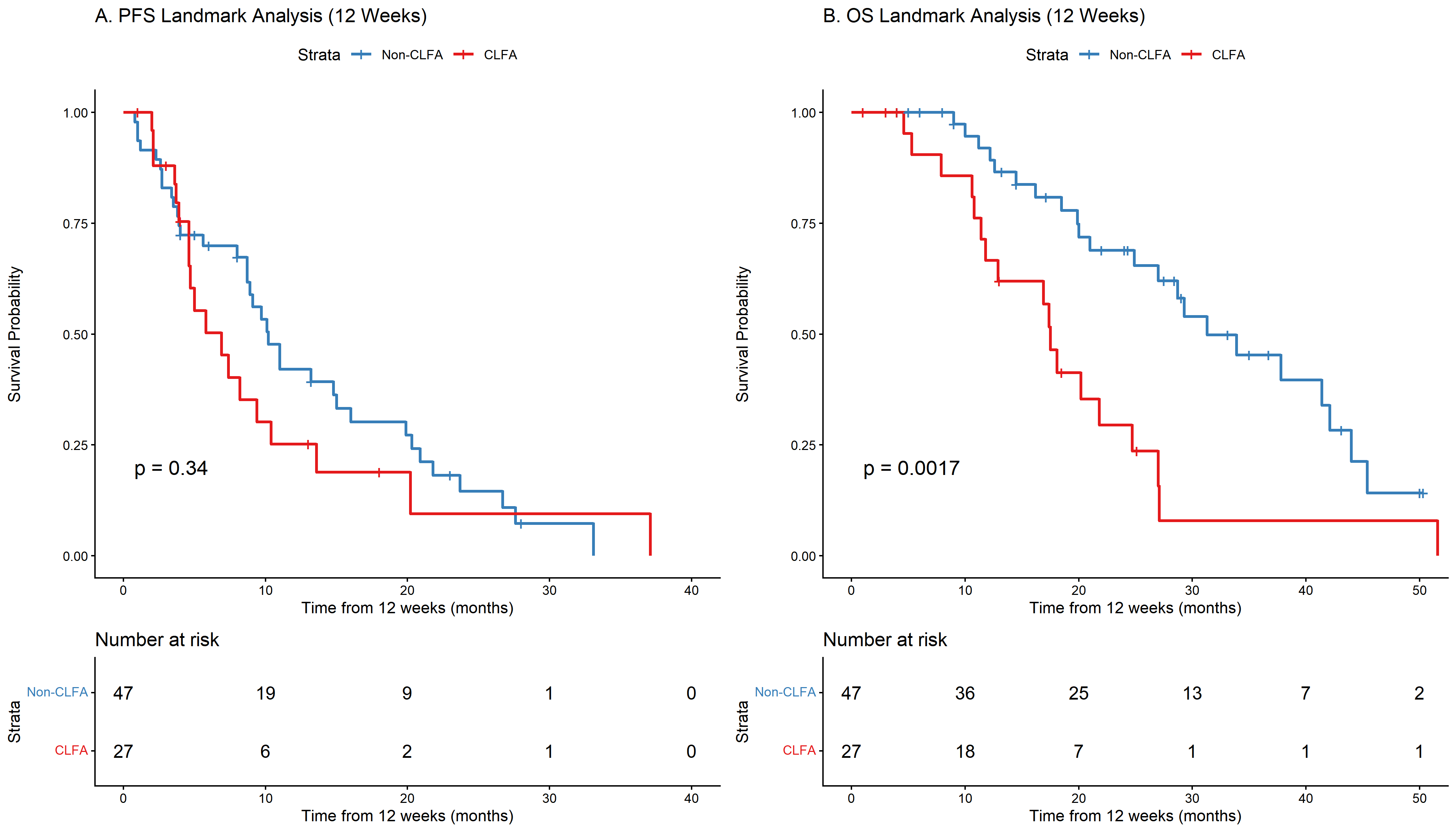

Supplement: Supplementary file 1 — Supplementary Material 1. [file 12885_2026_15616_MOESM1_ESM.png]

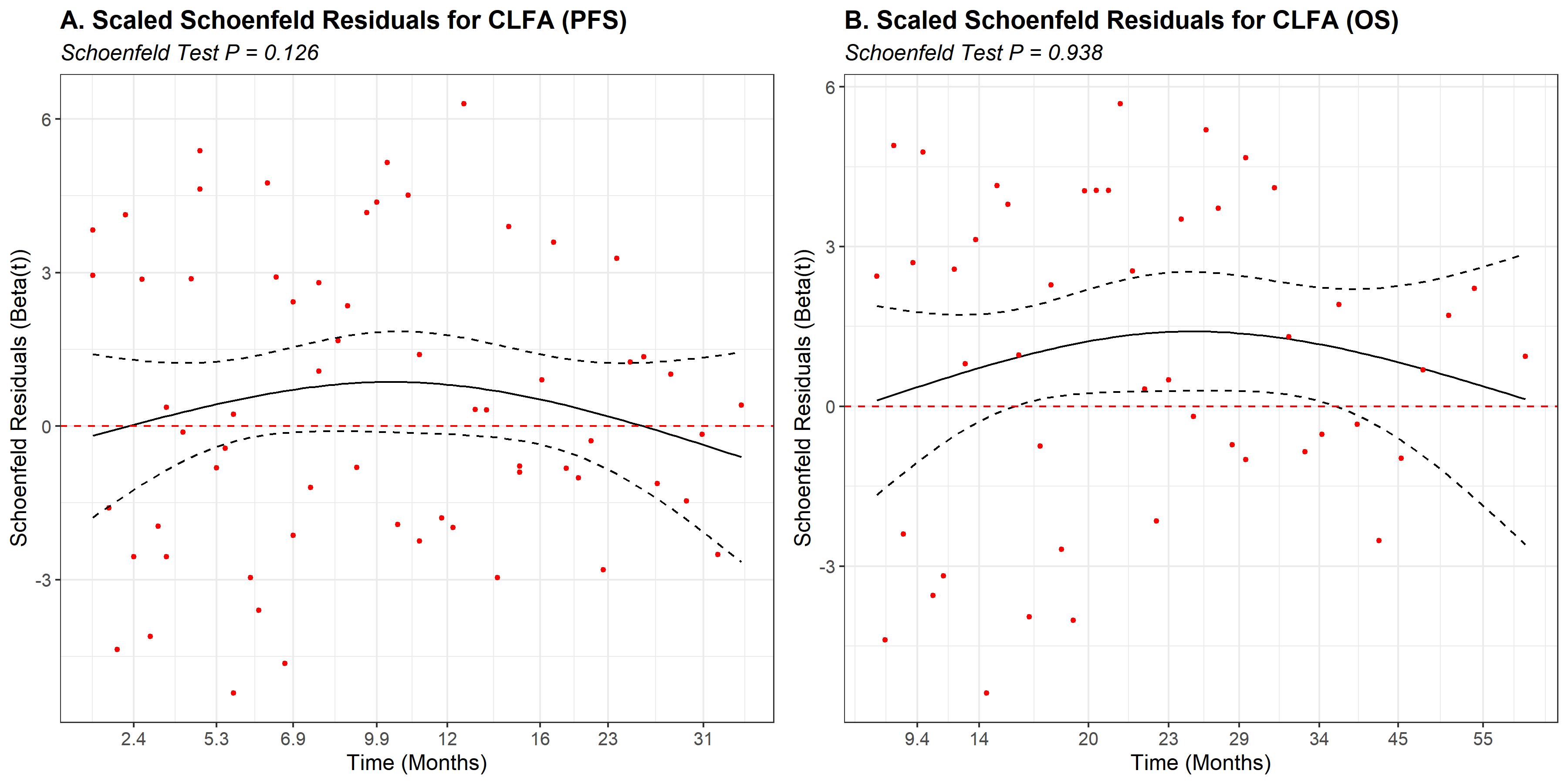

Supplement: Supplementary file 2 — Supplementary Material 2. [file 12885_2026_15616_MOESM2_ESM.png]

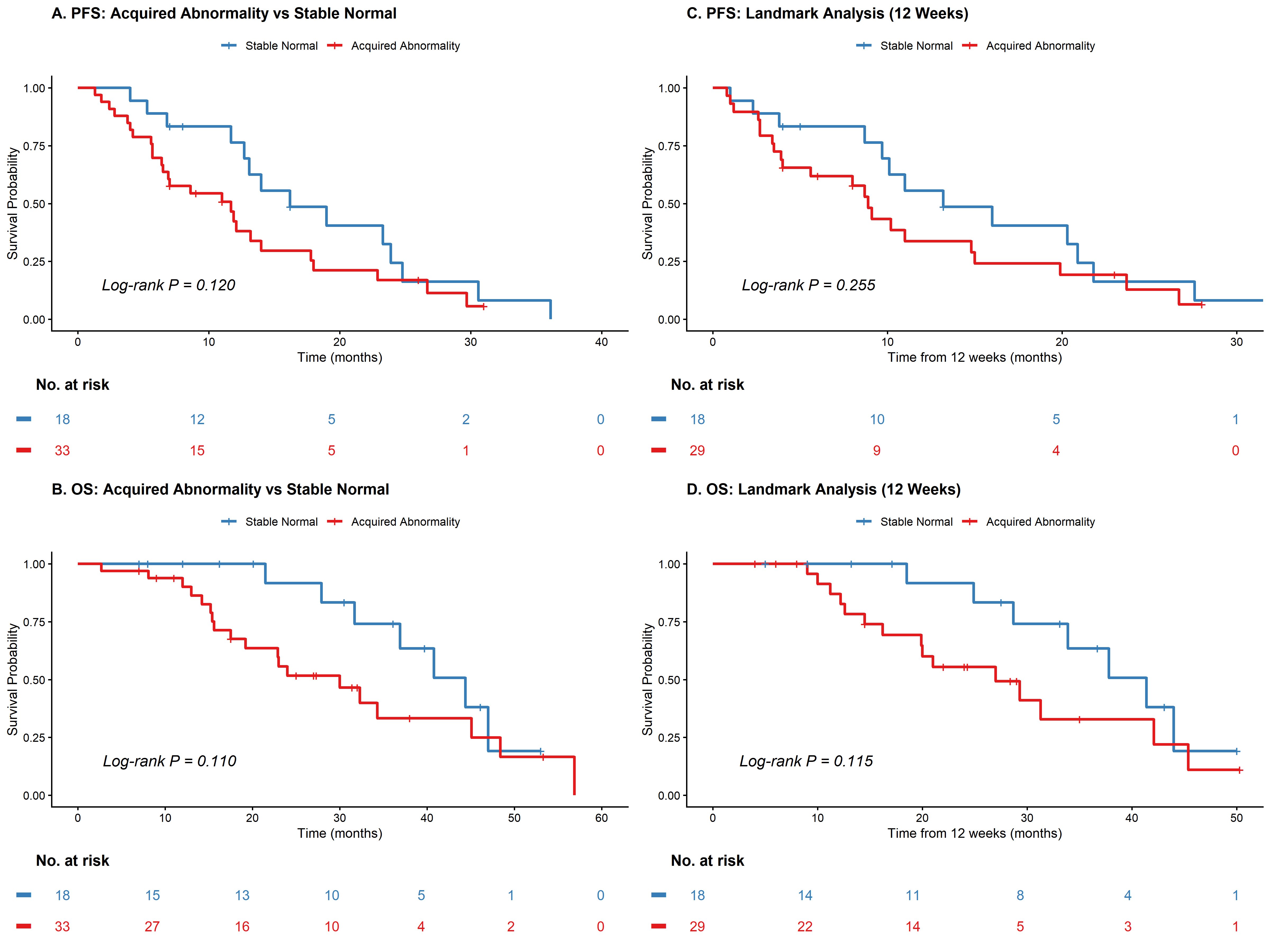

Supplement: Supplementary file 3 — Supplementary Material 3. [file 12885_2026_15616_MOESM3_ESM.png]
